# A two-in-one lignosulfonate carbon dots for bacterial detection and fluorescence quenching in food, pharmaceuticals, and cultural heritage preservation

**DOI:** 10.1038/s41598-025-28621-2

**Published:** 2025-12-10

**Authors:** Hebat-Allah S. Tohamy

**Affiliations:** Cellulose and Paper Department, National Research Centre, 33 El Bohouth Str, P.O. 12622, Dokki Giza, Egypt

**Keywords:** Lignosulfonate, Carbon dots, Bacterial detection, Luminescent sensors, Food safety, Pharmaceutical applications, Cultural heritage preservation, Biochemistry, Biological techniques, Biotechnology, Chemistry, Microbiology

## Abstract

A versatile, multifunctional nanocomposite based on lignosulfonate (LS) and carbon dots (CDs) was developed and characterized to demonstrate its dual capability for selective antimicrobial activity and differential microbial sensing. Fourier-transform infrared (FTIR) spectroscopy confirmed the successful synthesis of the LS-CDs, showing new characteristic peaks corresponding to N–H, C–N, and C–S bonds. Structural analysis indicated that the synthesis process led to a more uniform and tightly packed pore distribution (2.03–3.53 μm) compared to the pure LS, which enhanced the composite’s surface properties. Quantum chemical parameters from Density Functional Theory (DFT) calculations supported these findings, revealing that the LS-CDs possess a higher polarity (µ = 9.29 Debye) and a lower energy gap (E_g_​=0.0352 eV), signifying increased reactivity and a greater propensity for electron transfer. In biological assays, the LS-CDs exhibited no antimicrobial activity against the Gram-negative *Escherichia coli*, likely due to its protective outer membrane. However, the composite showed significant antimicrobial efficacy against the Gram-positive *Staphylococcus aureus* (16 mm inhibition zone) and the fungus *Candida albicans* (16 mm inhibition zone). This selective antimicrobial action is attributed to the strong electrostatic interactions between the LS-CDs and the simpler cell wall structures of these microorganisms, leading to cellular disruption. Furthermore, the LS-CDs demonstrated a remarkable differential sensing capability via unique fluorescence signals: a blue-to-red shift for *E. coli*, star-like green shapes for *S. aureus*, and red filaments for *C. albicans*. The observed fluorescence changes were accompanied by a sharp decrease in intensity from an initial value of 20.78 to final values of 6.46, 5.51, and 4.91, respectively, for *E. coli*, *S. aureus*, and *C. albicans*. This dual functionality positions the LS–CDs as a promising platform for applications in food safety, pharmaceutical quality control, and cultural heritage preservation.

## Introduction

Bacterial contamination is a pervasive and serious problem that affects nearly every aspect of our lives, from public health to industrial processes. Its severity stems from the potential for rapid proliferation and the wide range of harmful effects that can result. The consequences are particularly pronounced in key sectors such as food safety, healthcare, cultural heritage preservation and water management^1–6^. In the food industry, bacterial contamination poses a significant threat to public health and the economy. Foodborne illnesses, caused by pathogens like E. coli, Salmonella, and Listeria, lead to millions of hospitalizations and thousands of deaths each year globally. Beyond the human toll, there are substantial economic losses. Food spoilage due to bacterial growth costs the industry billions of dollars annually, encompassing product recalls, discarded goods, and damage to brand reputation^7–11^. Bacterial contamination in healthcare is a major driver of healthcare-associated infections (HAIs), which are a leading cause of morbidity and mortality worldwide^12^. These infections can be caused by bacteria like Methicillin-resistant Staphylococcus aureus and Clostridium difficile, which are particularly dangerous due to their antibiotic resistance^13,14^. Contamination can occur on medical instruments, in surgical suites, and on hospital surfaces, putting vulnerable patients at great risk^15,16^. The economic burden of HAIs is substantial, leading to longer hospital stays, increased treatment costs, and a greater need for specialized care^17,18^.

While not as obvious as its impact on health, bacterial contamination also poses a serious threat to cultural heritage. Microorganisms, including bacteria, can colonize and degrade a wide range of materials found in museums, archives, and historical sites, such as paper, textiles, wood, and stone^19,20^. These biodeterioration processes can cause irreversible damage, leading to discoloration, structural weakening, and the complete loss of invaluable artifacts and documents. Preserving these items requires a deep understanding of the bacteria involved and the environmental conditions that support their growth^21–23^. Furthermore, bacterial contamination is a critical issue in water management, threatening drinking water supplies and aquatic ecosystems. Waterborne pathogens like *Vibrio cholerae* and Giardia can cause severe diseases and lead to large-scale public health crises^24,25^. Inadequate water treatment and compromised infrastructure can allow these harmful microorganisms to enter the water supply, with devastating consequences. Ensuring the safety of drinking water requires continuous monitoring, effective disinfection processes (such as chlorination and UV treatment), and robust distribution systems^26^. Moreover, bacterial contamination from sources like agricultural runoff and industrial waste can harm aquatic life and disrupt ecosystems, impacting biodiversity and natural resource sustainability^27–29^. The traditional methods for detecting bacterial contamination, which often rely on laboratory culture techniques, can be slow and labor-intensive. This delay is a major problem, as it allows contaminated products to enter the supply chain before being identified^30–34^. Consequently, there’s a critical need for rapid, on-site detection methods that can quickly screen for bacterial presence at various points, from the farm to the processing plant and beyond. The development of new technologies, such as biosensors and microfluidic devices, is essential for ensuring a safer and more resilient food supply^7,34^.

Carbon dots (CDs) are a next-generation material that are rapidly gaining attention in a variety of scientific and technological fields. These novel nanomaterials are a promising alternative to traditional detection and imaging materials. Their key properties make them highly suitable for applications where safety and performance are paramount^11,32^. The mechanism for using CDs to detect bacteria is rooted in their tunable surface properties. By functionalizing their surfaces with specific molecules, CDs can be engineered to selectively bind to bacteria^33–36^. For example, a common strategy is to attach sulfur and nitrogen from thiourea source to the surface of the CDs. These molecules act as a fluorescent enhancers, where the sulfur, nitrogen–CDs (S, N–CDs) specifically recognizes and binds to a corresponding molecule on the bacterial cell wall. Once the S, N–CDs attaches to the bacteria, it can be detected using its strong fluorescence. When a sample containing bacteria is mixed with the functionalized CDs, the CDs will selectively bind to the bacteria, causing their fluorescence to either be quenched (turned off) or enhanced, providing a clear signal that indicates the presence of the bacteria^2,9^. The ability of CDs to rapidly and specifically detect bacteria makes them an ideal tool for various fields. In food safety, functionalized CDs can be incorporated into rapid test kits or biosensors to screen for common pathogens like *E. coli* and *Salmonella* directly at food processing factories or in shipping containers. This allows for quick, on-site testing, which can significantly reduce the risk of foodborne illness outbreaks. In the pharmaceutical industry, CDs can be used to monitor sterile environments and products^8,10^. Regular sampling and testing with CD-based sensors can provide a real-time assessment of microbial load, ensuring that manufacturing processes remain free from contamination and that sterile products meet the highest safety standards. The non-toxic nature of CDs is particularly beneficial here, as it minimizes the risk of introducing harmful substances into pharmaceutical products during testing^4,7,9^.

The unique aspect of this research is the use of lignosulfonate (LS) as a precursor for synthesizing CDs. While CDs can be made from a variety of carbon-rich materials, using LS is a novel approach that offers significant advantages in sustainability and cost-effectiveness. LS is a major byproduct of the pulp and paper industry, specifically from the process of making wood pulp^37–39^. This means it is an abundant, readily available, and low-cost raw material^39^. By utilizing this industrial waste product, our research aligns with the principles of green chemistry and waste valorization, which seek to transform waste into valuable resources. This approach reduces environmental burden and promotes a more sustainable, circular economy by finding new uses for materials that would otherwise be discarded. LS is an ideal precursor for the synthesis of CDs due to its inherent chemical structure. It possesses a rich aromatic structure and a high carbon content, which are essential for forming the carbon core of the dots^39^. These complex, pre-existing carbon rings in the LS molecule facilitate the formation of the desired fluorescent properties in the final CDs and this is also proved from Tohamy et al. who enhanced the fluorescent properties of CDs by adding a sulfur source from thiourea^9^. This makes the synthesis process more efficient and allows for the creation of CDs with robust and well-defined characteristics.

This manuscript presents a novel, cost-effective, and highly sensitive method for the detection of bacterial contamination using CDs derived from LS. Our specific goals include: synthesizing the lignosulfonate carbon dots (LS–CDs), thoroughly characterizing their chemical and optical properties, and demonstrating their selectivity and sensitivity toward a range of common bacteria, including *E. coli*, *S. aureus*, and *Candida albicans*. This work can be applied in the future to specific bacterial detection scenarios in the food, pharmaceutical, and cultural heritage fields.

##  Materials and methods

### Materials

Lignosulfonate (LS), NaOH and thiourea were sourced from were acquired from Sigma-Aldrich. We used all chemicals, reagents, and substrates as received, without further purification, and they were of analytical grade.

### Microwave assisted synthesis of lignosulfonate carbon dots brittle film

To synthesize lignosulfonate (LS-CDs), 15 g of LS was combined with NaOH solution, and 15 g of urea. This mixture which contains LS, NaOH, and thiourea were heated using microwave irradiation at 800 W for approximately 15 min. The newly synthesized LS-CDs were filtered and dried in Teflon plates to form brittle films. The LS mixture was used blank and denoted as LS.

### Characterization

Scanning electron microscopy (SEM) images were taken using a Quanta/250-FEG scanning electron microscope (Thermo Fisher Scientific, Waltham, MA, USA). The fluorescence microscopy was performed using a Jasco FP-6500 spectrofluorometer (made in Japan) with a 150-watt xenon arc lamp. Furthermore, the fluorescent intensity was calculated by Image J program. In addition, FTIR spectra were taken with a Mattson 5000 spectrometer (Unicam, United Kingdom) using the potassium bromide (KBr) disk method. The crystallinity index (LOI) was calculated using the Eqs. (1) and (2).

1 $$\text{LOI} = \:\frac{{A}_{1425}}{{A}_{900}}$$

2 $${\text{MHBS}} = \:\frac{{A}_{OH}}{{A}_{CH}}$$

where A_1425_ and A_900_ refer to the FTIR absorbance at 1425 and 900 cm^− 1^, respectively. In addition, A_OH_ and A_CH_ refer to the FTIR absorbance of the OH and CH peaks, respectively^40–48^.

DFT calculations using the B3LYP/6-31G(d) level of theory with Berny optimization were performed using Gaussian 09 W. Investigated parameters included optimized geometries, ground state energies (E_T_), HOMO−LUMO energies (EHOMO, ELUMO, and E_g_), dipole moment (µ), absolute hardness (η), softness (σ, S), and additional electronic charge (ΔN_max_)^49–51^.

3 $$\:\:{E}_{gap}=({E}_{LUMO}-{E}_{HOMO})$$

4 $$\:{\upeta\:}=\frac{({E}_{LUMO}+\:{E}_{HOMO})\:\:}{2}\:$$

5 $$\:{\upsigma\:}=\frac{1\:\:}{{\upeta\:}}$$

6 $$\:\text{S}=\frac{1\:\:}{2{\upeta\:}}$$

7 $$\:{\Delta\:}{N}_{max}=\frac{-\text{P}\text{i}\:\:}{{\upeta\:}}$$

## Results and discussion

### Sensing study against microbial pollution

**Fig. 1:**
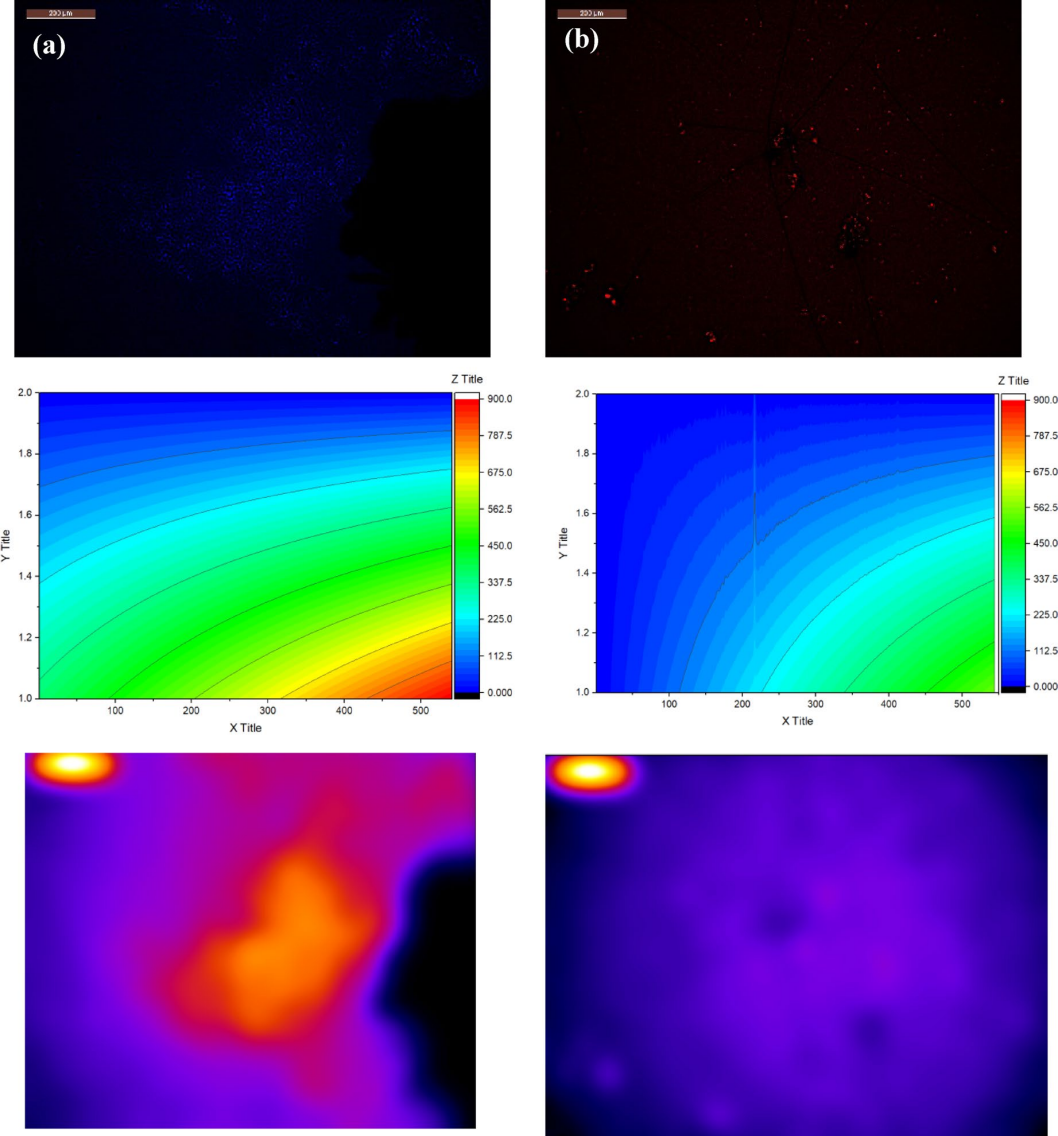

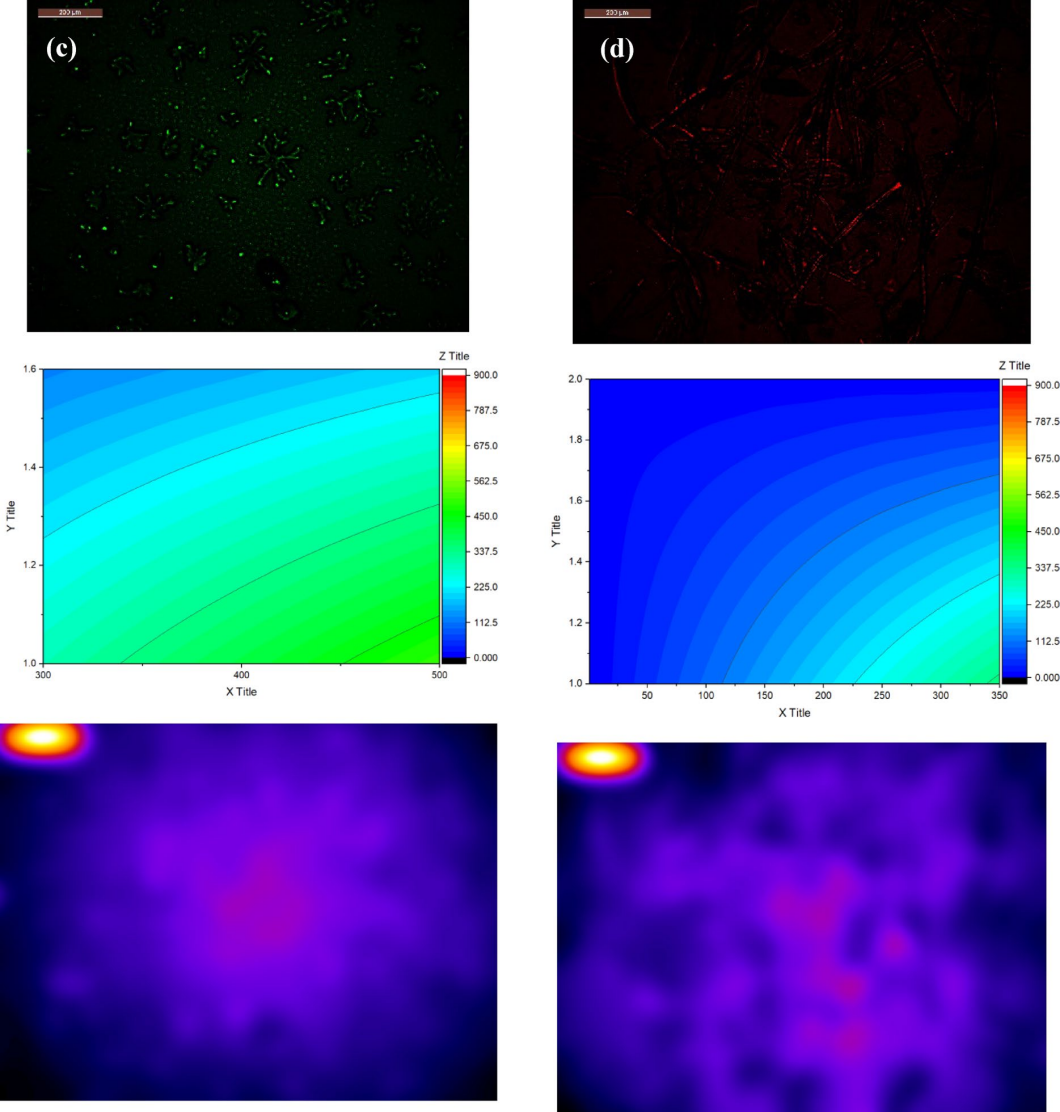
Fluorescence microscope with contour plots and density scatter for (a) LS–CDs before bacterial contact, (b) LS–CDs/*Escherichia coli*, (c) LS–CDs/*Staphylococcus aureus*, and (d) LS–CDs/*Candida Albicans*.

The observed color shift in fluorescence from blue to red is a bathochromic shift, and a dramatic drop in intensity from 20.78 to 6.46 that strongly suggests a specific chemical or physical interaction between the LS–CDs and the *E. coli* cellular components (Fig. 1). This interaction likely involves adsorption of the LS–CDs onto the bacterial cell wall, or specific binding to surface proteins or lipids. This provides a powerful method for sensing or detecting the presence of bacteria. However, the lack of antimicrobial activity in the same test is a critical finding. An antimicrobial effect requires a mechanism that is destructive enough to inhibit bacterial growth or cause cell death, such as membrane disruption, inhibition of key enzymes, or oxidative stress. The interaction observed via fluorescence microscopy, while a clear signal, is likely non-lethal. The LS–CDs may be binding to the cell surface without causing sufficient damage to the cell membrane, or their concentration and surface properties may not be optimized for a lethal effect. In contrast, the interaction with *Staphylococcus aureus* led to a star like shapes with green color and a similar drop in intensity, from 20.78 to 5.51, which is accompanied by an antimicrobial effect. Finally, when exposed to the fungus *C. albicans*, the composite forms distinct red filaments with an intensity decrease from 20.78 to 4.91, also leading to cell death. The material demonstrates a dual function:

1. Sensing: It can identify different microbes based on unique fluorescence signals without necessarily killing them (i.e. *E. Coli*, it will be discussed after).
2. Antimicrobial action: It selectively inhibits Gram-positive bacteria (i.e. *S. aureus*) and fungi (i.e. *Candida albicans*, it will be discussed after).

This dual functionality makes LS−CDs promising for various applications, including food safety, pharmaceutical quality control, and cultural heritage preservation. The distinct interactions with different microorganisms are due to their unique cell wall structures, with the complex outer membrane of Gram-negative bacteria like *E. coli* acting as a barrier against the LS–CDs’. This dual functionality can be explained by the fundamental differences in cell wall structure between these microorganisms. The complex outer membrane of Gram-negative *E. coli* likely prevents the LS–CDs from accessing and disrupting the inner cell membrane. As a result, the LS–CDs only cause a surface-level interaction that triggers a fluorescence signal without causing cell death. In contrast, the simpler cell wall of Gram-positive *S. aureus* and the different cell envelope of fungal *C. albicans* are more susceptible, allowing the LS-CDs to interact directly with the cell membrane or internal components and exert their cytotoxic effect. This unique combination of properties has significant implications for several applications. In food safety, the LS–CDs can provide a powerful, one-step solution. They can be applied to packaging or surfaces to rapidly detect the presence of indicator bacteria like *E. coli* while simultaneously eliminating harmful Gram-positive bacteria and fungi that can cause spoilage or illness. This offers both a real-time warning system and an active layer of protection. Similarly, in the pharmaceutical industry, this technology is invaluable for quality control. A single test could be used to verify the absence of *E. coli* while also ensuring the sterilization of the product from other common contaminants like *S. aureus* and *C. albicans*. This streamlines the quality assurance process, making it faster and more comprehensive. For cultural heritage preservation, the LS-CDs offer a new, non-destructive method for both monitoring and treatment. The LS–CDs’ antimicrobial properties would then work to eliminate harmful fungi and bacteria without the need for additional chemical treatments, ensuring the long-term preservation of priceless artifacts. Overall, the findings demonstrate that the LS-CDs are a versatile, multifunctional material capable of sophisticated and selective interactions with a variety of microorganisms, making them a promising candidate for a wide range of scientific and commercial applications.

The shift in the contour plots indicates that the distribution of a specific property, likely the fluorescence signal has changed. Before contact with bacteria, the fluorescence signal was likely spread out, but after interaction, it became concentrated in a specific region, which is corroborated by the density scatter plot. The density scatter plot reinforces this finding. The fact that the density became “concentrated in the top left region” suggests that the LS–CDs are aggregating or binding specifically to the bacterial cells. This high concentration of a signal in a specific area supports the idea of targeted binding. The LS–CDs are not simply dispersed randomly; they are accumulating on the surface of or around the bacteria.

### PCA plot with FTIR and SEM analysis

**Fig. 2:**
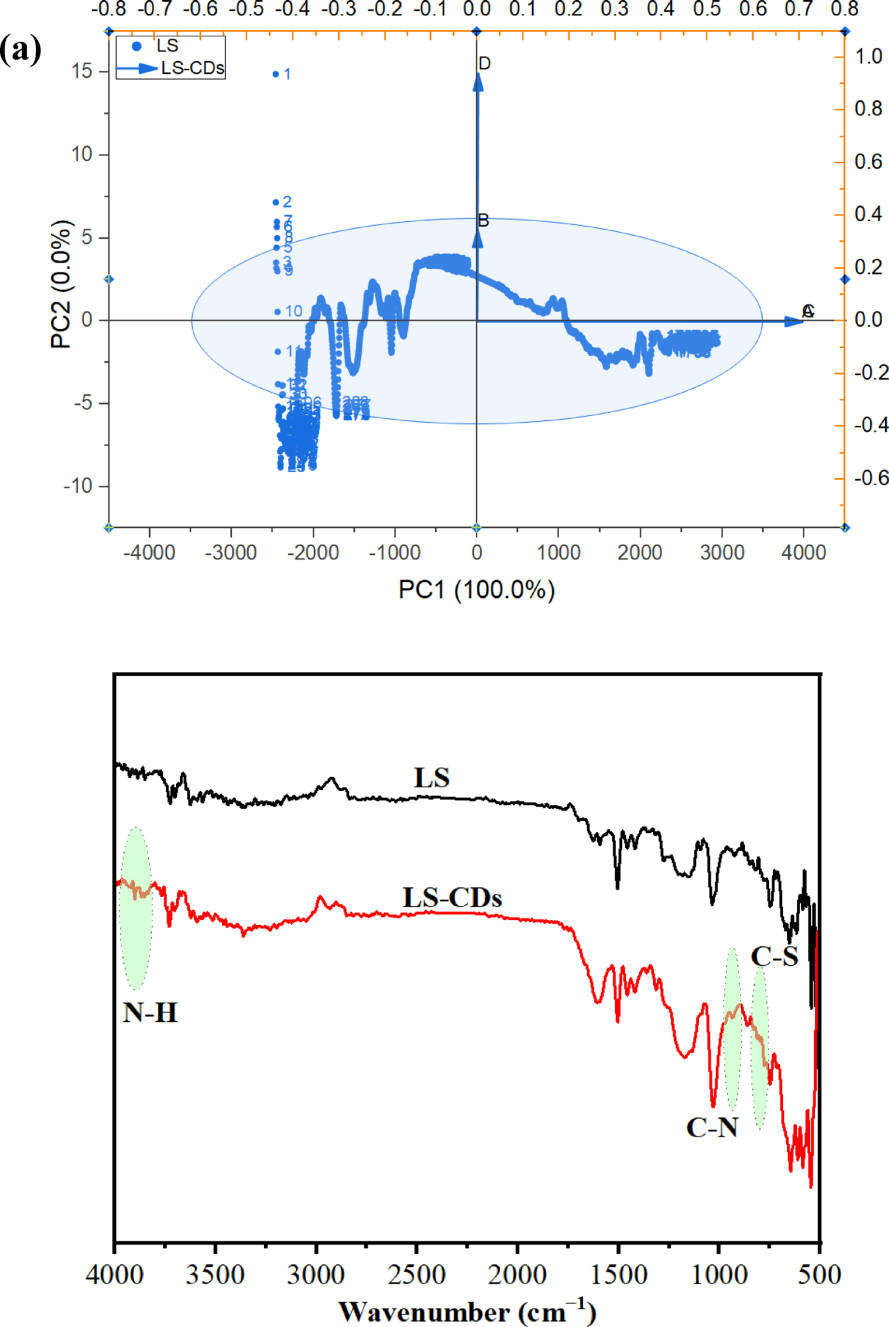

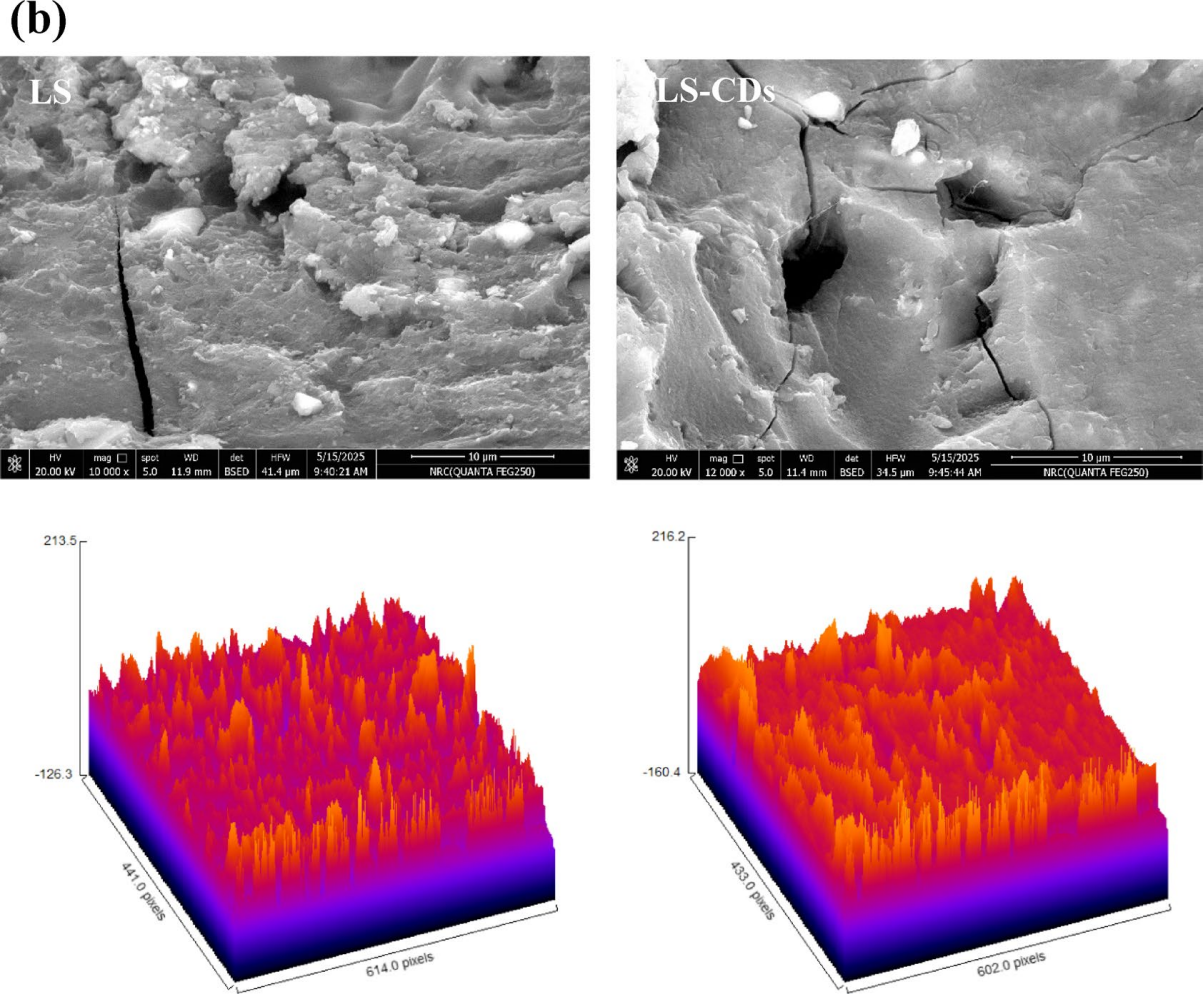
(a) PCA plot with FTIR spectra, and (b) SEM analysis for LS and LS–CDs with 3D view.

FTIR spectra of the LS (Fig. 2a) is characterized by the following absorbance peaks: 651 cm^–1^ (-CH_2_ bonds associated with the aromatic ring); 1035 cm^– 1^ (–CH_3_ groups); 1276 cm^–1^ (–SO_3_ groups); 1417, 1504, and 1594 cm^–1^ (substituted lignin aromatic ring); 1625 cm^–1^ (C = C); 1697 cm^− 1^ (vibrations characteristic to -C = O groups of aromatic acids); 3725 cm^–1^ (–OH)^52,53^. The LS–CDs showed the dame peaks with additional peaks at 3901 cm^–1^ (N–H); 862 cm^–1^ (C–N); and 779 cm^[–1^ (C–S), which prove the S, N–CDs preparation (Fig. 2a)^9,11,33^.

The pore size from pure LS to the LS–CDs composite reveals a key structural change resulting from the synthesis process (Fig. 2b). The pure LS exhibited a pore size range of 1.73–3.92 μm. After microwave treatment in the presence of thiourea and NaOH, the resulting CDs composite showed a different pore size distribution of 2.03–3.53 μm. This indicates that the preparation process caused a redistribution of pore sizes, resulting in the elimination of the largest pores (> 3.53 μm) and the smallest pores (< 2.03 μm) present in the pure LS. This transformation likely leads to a more uniform and tightly packed structure. The presence of thiourea and NaOH during the synthesis plays a crucial role in this morphological change. The thiourea acts as a sulfur and nitrogen source for doping and a pore-forming agent. The high-temperature microwave process causes the thiourea to decompose, releasing gases that can influence the formation of new pores and restructure existing ones. The NaOH, as a strong base, can catalyze the breakdown of the lignosulfonate polymer chains. This breakdown and subsequent reassembly of the smaller molecules around the newly forming carbon dots leads to a more compact and ordered structure. The removal of the largest pores suggests that the new, smaller LS–CDs fill these voids, while the elimination of the smallest pores may be due to the expansion of micro- or mesopores during the high-temperature synthesis. This structural modification is a key reason for the enhanced performance of the LS–CDs composite, as a more uniform pore distribution and higher surface area can significantly improve adsorption and sensing capabilities.

### Molecular electrostatic potential mapping and DFT calculations

**Fig. 3:**
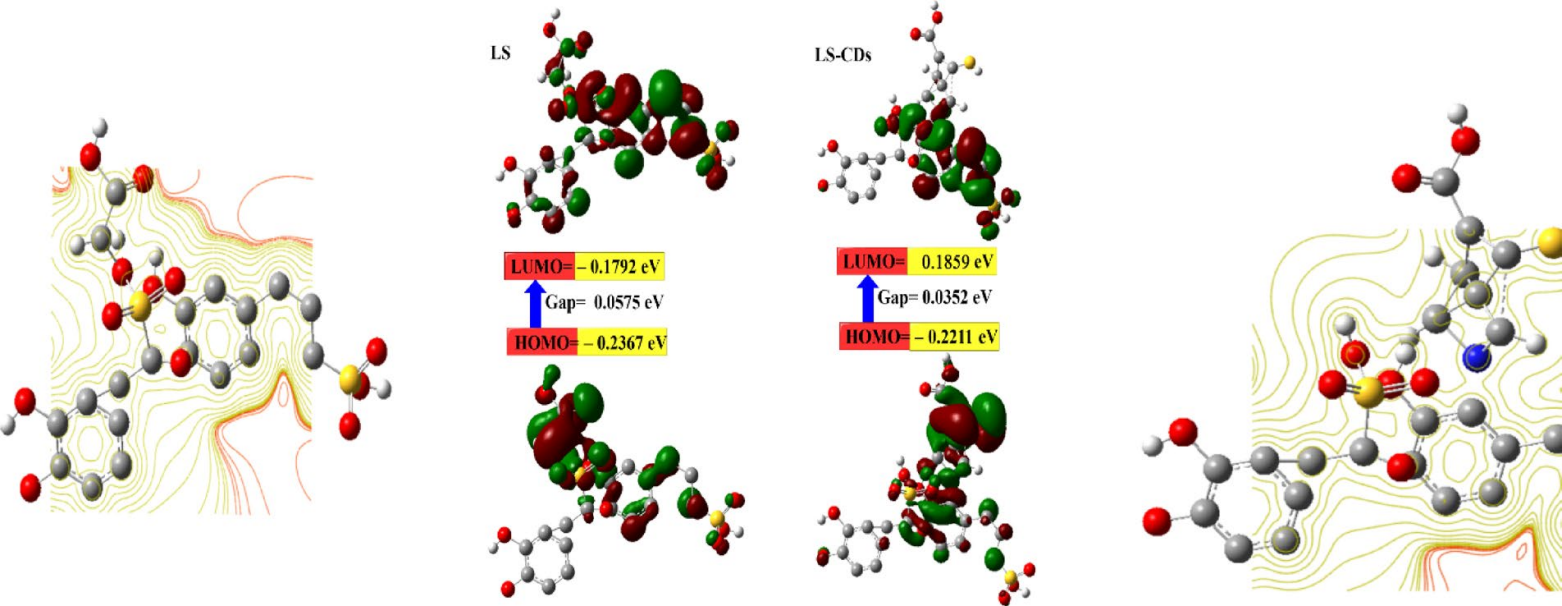
The ESPM and the HOMO–LUMO gap energies for the LS and LS–CDs.

The high µ for LS–CDs (9.29 Debye) compared to LS (6.53) is an indication of the strong sensitivity^35,49^. This was also confirmed from the lower number of the calculated E_g_ for the LS–CDs (0.0352 eV). The µ is a measure of the overall polarity of a molecule, calculated as a vector sum of all individual bond dipoles (Fig. 3; Table 1). A high µ for the LS–CDs indicates a large separation of positive and negative charge across the entire structure. This can be caused by the synthesis process, which breaks down the original LS polymer and creates new, highly functionalized, and asymmetric nanostructures. These new structures possess an uneven distribution of electron-withdrawing and electron-donating groups, leading to a greater net µ for the molecule as a whole. In contrast, the Molecular Electrostatic Potential map (ESPM) visualizes the charge distribution on the surface. Red regions represent areas of high electron density (negative charge). The observation of a lower red intensity for the LS–CDs suggests that while the overall molecule is more polar (higher µ), the specific negative charge is less concentrated in one or a few localized “hot spots” on the LS–CDs surface. Instead, the charge is likely more dispersed or spread out over the new, larger surface area of the nanostructure. This is a result of the chemical reactions with urea and NaOH, which react with the oxygen-rich sites on the lignosulfonate, breaking up the strong electron localization^49^. From DFT, the increase in the ω value for LS–CQDs (–0.1017 eV) compared to LS (–0.1040 EV) is an evidence of strong energy changes between the donor (HOMO) and acceptor (LUMO)^10,51^. The E_T_ for the LS–CDs (–3065.59 au) is lower than that of the LS (–2408.39 au), meaning is stability and strong bonding. The S value for LS–CDs (–2.457 eV) is lower than LS (–2.404 eV) indicates a higher rigidity and lower mechanical properties due to a lack of elasticity^2,7,8^.

**Table 1:** The quantum chemical parameters of LS and LS–CDs.

DFT B3LYP/6–31G (d)	LS	LS–CDs
E_LUMO_ (eV)	–0.1792	–0.1859
E_HOMO_ (eV)	–0.2367	–0.2211
E_g_ (eV)	0.0575	0.0352
E_T_ (au)	–2408.39	–3065.59
µ (Debye)	6.53	9.29
ɳ (eV)	–0.2079	–0.2035
σ (eV)	–4.8097	–4.9140
S (eV)	–2.404	–2.457

### Mechanism of fluorescence quenching

Fluorescence quenching can occur through several mechanisms. Photoinduced electron transfer (PET) is a particularly relevant mechanism here. In PET, an electron is transferred from the excited LS–CDs to a nearby molecule (the bacteria’s biomolecules), or vice versa. This non-radiative process de-excites the fluorophore (i.e. LS–CDs), preventing it from emitting light and causing the observed intensity drop^54,55^. The DFT calculations which discussed above support this, as they indicate the LS–CDs have a low E_g_ and a high electron-accepting capacity (ω), making them highly susceptible to electron transfer with microbial components. Low E_g_ confirms the LS–CDs’ high reactivity which is the fundamental precursor for potential mechanisms like ROS generation or PET, leading to cellular damage. Furthermore, the quenching effect could also involve dynamic quenching. Dynamic quenching involves a collision between the excited LS–CDs and the quencher (i.e. bacteria), leading to a non-radiative energy transfer. The dramatic decrease in intensity suggests a very efficient quenching process, which is an ideal characteristic for a highly sensitive sensor.

###  Antimicrobial mechanism

**Fig. 4:**
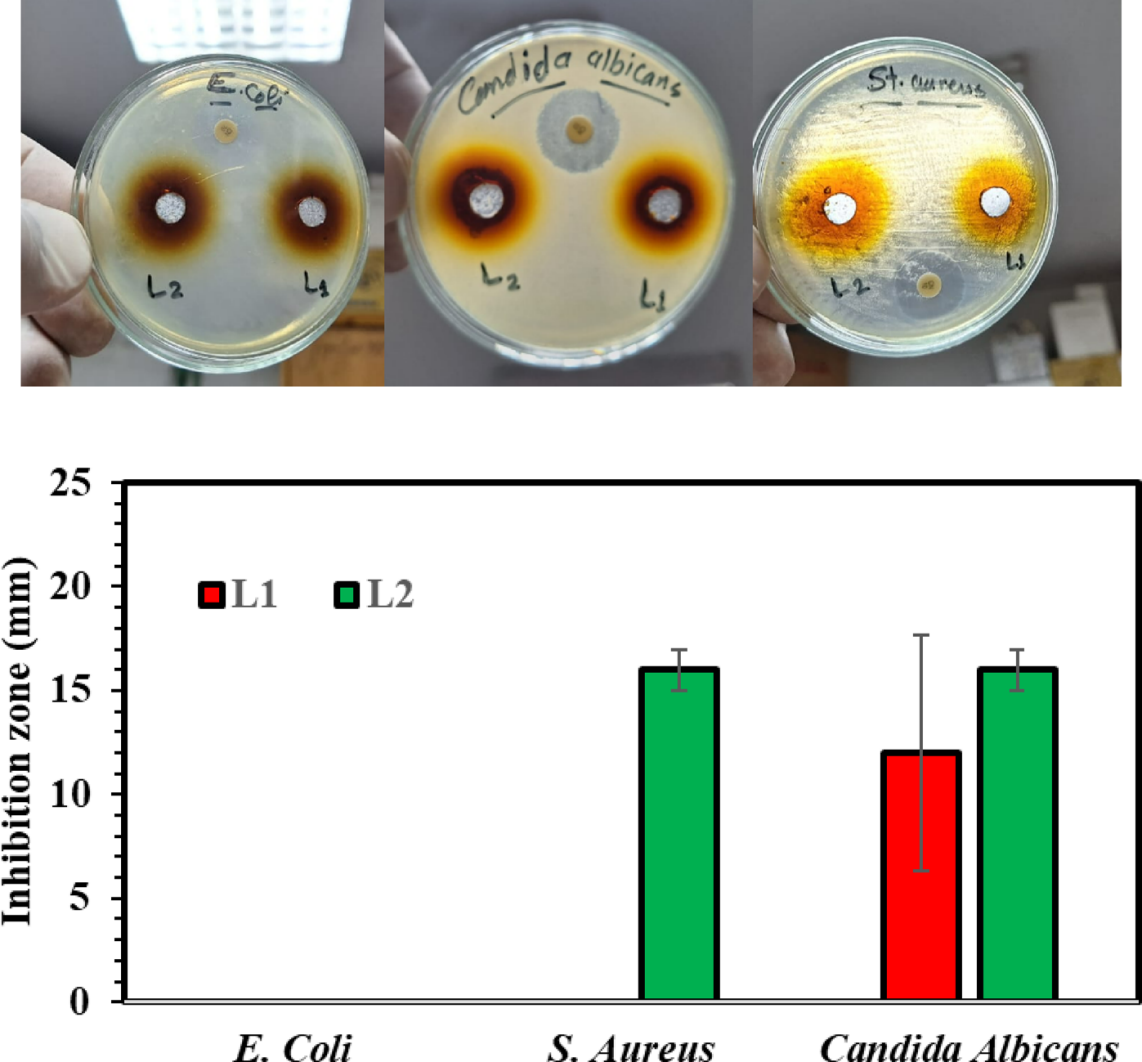
Antimicrobial activity of the LS (denoted as L1) and LS–CDs hydrogel (denoted as L2) with standard deviation error bars.

The antimicrobial results showed that both the LS (L1) and the LS–CDs (L2) demonstrated no antibacterial activity against *Escherichia coli*. This lack of activity is likely due to the highly effective outer membrane of *E. coli*, which is rich in lipopolysaccharides (LPS) and acts as a robust barrier against the ingress of larger or less-active compounds (Fig. 4). In addition, LS displayed no inhibition zone for *Staphylococcus aureus*, whereas LS–CDs exhibited an inhibition zone of approximately 16 mm. This enhanced activity is attributed to the presence of the CDs, which possess unique surface properties and functional groups. These features likely facilitate stronger interactions, such as electrostatic attraction, with the negatively charged teichoic acids on the surface of Gram-positive bacteria, leading to a disruption of the cell wall. Furthermore, the LS–CDs composite demonstrated superior antifungal activity against *Candida Albicans*, with an inhibition zone of 16 mm, compared to the 12 mm inhibition zone observed with pure LS. This improvement in both antibacterial and antifungal activity underscores the crucial role of the CDs in enhancing the antimicrobial properties of the LS matrix, likely through a combination of physical and chemical interactions that are ineffective against the Gram-negative cell barrier. The clear empirical observation of killing Gram-positive (*S. aureus*) and fungi (*C. albicans*) but not Gram-negative (*E. coli*) is the primary evidence for the mechanism. We have elaborated that the *E. coli* outer membrane (LPS layer) serves as an impenetrable physical barrier that prevents the highly polar LS–CDs from reaching the cell membrane, regardless of the CDs’ high reactivity. This unique Gram-negative barrier is the key factor confirming the proposed electrostatic/surface-interaction mechanism.

The strong electronic properties of the LS–CDs, as confirmed by DFT calculations, directly correlate with their enhanced antimicrobial activity. The high µ of the LS–CDs, indicative of greater molecular polarity, facilitates stronger electrostatic attraction to the negatively charged microbial cell surfaces, such as the teichoic acids in *Staphylococcus aureus*. This strong binding is the first step in the antimicrobial mechanism. Furthermore, the significantly lower E_g_ of the LS–CDs suggests a higher reactivity and a greater ease of electron transfer. This property enables the CDs to interact more readily with essential microbial components and potentially generate reactive oxygen species (ROS), leading to oxidative stress and cellular damage. The increased ω further confirms this, showing that the LS–CDs are more prone to accepting electrons from microbial proteins or other vital biomolecules, disrupting their function and ultimately causing cell death^11,32,33^. In essence, the favorable electronic and structural characteristics determined by DFT provide a solid theoretical explanation for the observed antimicrobial efficacy of the LS-CDs composite.

### Versatile applications of LS–CDs

#### Application in food safety

Bacterial contamination in food requires both rapid identification and active spoilage prevention. The LS–CDs provides a comprehensive, two-tiered defense:

- Real-time warning system (sensing): The non-lethal interaction with Gram-negative organisms like *E. coli* is critical. While *E. coli* is a common hygiene indicator, its complex LPS layer prevents killing, leading to the distinct blue-to-red fluorescence shift and signal quenching. This offers a rapid, non-destructive optical signal that warns of potential fecal contamination or hygiene breaches before pathogens proliferate.
- Active spoilage prevention (killing): The LS–CDs actively eliminates common spoilage agents and pathogens. The robust efficacy demonstrated against Gram-positive *S. aureus* (16 mm zone) and fungus *C. albicans* (16 mm zone) means the material can be integrated into smart packaging or contact surfaces to actively extend shelf life and neutralize major causes of foodborne illness.

The LS–CDs material offers both a real-time diagnostic tool and an active protective layer, streamlining food quality assurance.

#### Application in pharmaceutical quality control

Ensuring the sterility of pharmaceutical products and environments is paramount, requiring rapid screening for a variety of microbial contaminants.

- Comprehensive quality assurance: The LS–CDs can simplify quality control protocols by simultaneously detecting and neutralizing common contaminants in a single assay.
- Differential detection: The material provides a unique optical signature for the three microbial types tested:
  - Gram-negative indicators (*E. coli*): A rapid non-lethal blue-to-red shift to confirm the presence of organisms that breach sterile barriers.
  - Gram-positive bacteria (*S. aureus*): Immediate detection via the star-like green shapes.
  - Fungal contamination (*C. albicans*): Detected by the unique red filaments, ensuring the product is free of common fungal threats.

The ability of LS–CDs to combine detection and sterilization makes this platform invaluable for validating cleanroom environments and maintaining product integrity.

#### Application in cultural heritage preservation

The use of LS–CDs offers a novel, non-destructive methodology for both monitoring and remediation of artifacts susceptible to biodeterioration, e.g., textiles, paper, wood (Table 2).

- Real-time, non-destructive monitoring (sensing function):
  - A minute, localized application of the LS–CDs dispersion to a suspect area allows the conservator to immediately determine the nature of the threat under a portable fluorescence microscope.
  - The unique signals—distinct red filaments for *C. albicans* and star-like green shapes for *S. aureus*—provide instant diagnostic feedback. This eliminates the delay and sample destruction associated with conventional laboratory culture techniques.
- Targeted, non-aqueous remediation (antimicrobial function):
  - The LS–CDs’ broad efficacy (16 mm inhibition zones) against common heritage biodeterioration agents (Gram-positive bacteria and fungi) allows for targeted neutralization of the identified threat.
  - The treatment is chemically superior to many traditional conservation biocides because the LS–CDs are derived from a sustainable, lignin-based precursor, offering a potentially lower environmental impact and reduced risk of staining or long-term chemical degradation to the priceless artifact.

**Table 2:** Versatile applications in food safety, pharmaceuticals, and cultural heritage preservation.

Application sector	Findings	Thematic clarity point
Food safety	Selective killing (*S. aureus*, *C. albicans*) + differential sensing (*E. coli*).	The LS–CDs offer a two-tiered protection: active spoilage prevention (killing Gram-positives/fungi) and a warning system (fluorescence signal for Gram-negatives like *E.coli*, a common hygiene indicator).
Pharmaceutical quality control	Differential sensing (*E. coli*) + broad killing (*S. aureus*, *C. albicans*).	Enables a single, rapid quality assay. It ensures sterility by eliminating Gram-positives and fungi while simultaneously providing an immediate, non-lethal confirmation of contamination by Gram-negatives (often sterile environment indicators), streamlining QA.
Cultural heritage preservation	Differential sensing (unique shapes) + antifungal/antibacterial efficacy.	Provides a non-destructive monitoring and remediation tool. Conservators can use the unique fluorescence signatures (color shift) for instant, non-invasive identification of specific biodeterioration threats (the “sensing”), followed by targeted local treatment (the “antimicrobial action”).

## Conclusions

The LS–CDs are a highly promising multifunctional material. FTIR and DFT analysis confirmed that the synthesis process successfully created LS–CDs, which possess enhanced electronic and structural properties compared to pure LS. The presence of new peaks at 3901 cm⁻¹ (N-H), 862 cm⁻¹ (C-N), and 779 cm⁻¹ (C-S) in the LS–CDs FTIR spectra confirms the successful incorporation of sulfur and nitrogen atoms. This chemical modification, along with a redistribution of pore sizes, creates a more compact and uniform structure, improving the composite’s overall properties. The enhanced properties of the LS–CDs, as supported by DFT calculations, directly correlate with their increased biological activity. The LS–CDs’ high polarity (µ) and low energy gap (E_g_​) make it highly reactive and capable of strong electrostatic interactions with negatively charged microbial cell surfaces, leading to cell death in susceptible organisms. Most importantly, the LS–CDs demonstrates a dual functionality: it can act as both a selective antimicrobial agent and a real-time sensing platform. The material effectively kills Gram-positive bacteria like *S. aureus* and fungi like *C. albicans*, as shown by the large inhibition zones (16 mm). However, it shows no lethal effect on Gram-negative *E. coli* due to the barrier of its complex outer membrane. This non-lethal interaction with *E. coli* is not without purpose; it triggers a distinct fluorescence change (blue-to-red shift with a dramatic intensity drop), allowing for the rapid detection of the bacterium. This unique combination of selective antimicrobial activity (against *S. aureus* and *C. albicans*) and differential fluorescence-based sensing (of *E. coli*) makes the LS–CDs a versatile platform with significant potential, specifically enabling real-time spoilage warning in food safety, streamlined quality control in pharmaceuticals, and non-destructive monitoring and treatment in cultural heritage preservation.

## Data Availability

All data generated or analysed during this study are included in this published article.
